# Effectiveness and safety of monoclonal antibodies for metastatic colorectal cancer treatment: systematic review and meta-analysis

**DOI:** 10.3332/ecancer.2015.582

**Published:** 2015-10-15

**Authors:** Bruno Rosa, Jose Paulo de Jesus, Eduardo L de Mello, Daniel Cesar, Mauro M Correia

**Affiliations:** Instituto Nacional de Câncer, Rio de Janeiro 20230-130, Brazil

**Keywords:** monoclonal antibody, cetuximab, bevacizumab, panitumumab, colorectal neoplasia, metastatic colorectal cancer

## Abstract

**Background:**

The effectiveness of chemotherapy (CT) for select cases of metastatic colorectal cancer (MCRC) has been well established in the literature, however, it provides limited benefits and in many cases constitutes a treatment with high toxicity. The use of specific molecular biological treatments with monoclonal antibodies (MA) has been shown to be relevant, particularly for its potential for increasing the response rate of the host to the tumour, as these have molecular targets present in the cancerous cells and their microenvironment thereby blocking their development. The combination of MA and CT can bring a significant increase in the rate of resectability of metastases, the progression-free survival (PFS), and the global survival (GS) in MCRC patients.

**Objective:**

To assess the effectiveness and safety of MA in the treatment of MCRC.

**Methods:**

A systematic review was carried out with a meta-analysis of randomised clinical trials comparing the use of cetuximab, bevacizumab, and panitumumab in the treatment of MCRC.

**Results:**

Sixteen randomised clinical trials were selected. The quality of the evidence on the question was considered moderate and data from eight randomised clinical trials were included in this meta-analysis. The GS and PFS were greater in the groups which received the MA associated with CT, however, the differences were not statistically significant between the groups (mean of 17.7 months versus 17.1 months; mean difference of 1.09 (CI: 0.10–2.07); *p* = 0.84; and 7.4 versus 6.9 months. mean difference of 0.76 (CI: 0.08–1.44); *p* = 0.14 respectively). The meta-analysis was not done for any of the secondary outcomes.

**Conclusion:**

The addition of MA to CT for patients with metastatic colorectal cancer does not prolong GS and PFS.

## Introduction

Colorectal cancer (CRC) is the third most common type of malignant neoplasia diagnosed in men and women in the world, and is also the second principal cause of death from cancer in the United States, following only lung cancer [1–7]. In Brazil, according to data from the National Cancer Institute (INCA), the estimates for the year 2014 indicate the occurrence of 15,070 new cases of CRC in men and 17,530 in women. These figures correspond to an estimated risk of 15.44 new cases in each 100,000 men and 17.24 in each 100,000 women, constituting the fourth most common absolute type of cancer in the Brazilian population [8].

CRC develops slowly, over several years of progression through different molecular and cytological stages, becoming a carcinoma with the potential for invasion and metastasis [4, 9]. Metastasis to the liver is the second most common (the first is to the lymph nodes) and affects approximately half of the patients with CRC, where only 11.7% survive for about five years following the diagnosis [7, 10, 11]. Surgical resection continues to be the only procedure which provides a possible cure [12], however, less than 20% of patients have liver metastases of colorectal tumours (LMCRC) which are potentially resectable [13].

For several decades, the basis for systemic treatment of advanced LMCRC has been chemotherapy (CT), composed of 5-fluorouracil (5-FU) in combination with leucovorin (LV), for which the therapeutic result has been the achievement of a global survival (GS) of 12 months [7, 10]. More recent agents, such as irinotecan (IR) and oxaliplatin (OX) improve the response and survival rates [7, 10, 14, 15]. It is well established in the literature that the administration of pre-operative CT to patients with initially non-resectable LMCRC can reduce the size of the tumours such that curative resection becomes possible (conversion chemotherapy), and can also reduce the risk of recurrence following resection. In addition, it helps to predict the prognosis by determining the tumour response, which in the end helps to exclude non-responders and select the best adjuvant therapy [5, 12]. Randomised clinical trials and meta-analyses have shown improvement in the response and survival rates with first-line chemotherapy (5-Fu/LV) in patients with LMCRC [7, 12, 16], such as the addition of oxaliplatin to this protocol (FOLFOX) significantly improves the response rate and progression-free survival (PFS) in advanced cases of LMCRC [7].

Meanwhile, a phase III study of the European Organisation for Research and Treatment of Cancer (EORTC) concludes that pre-operative CT (FOLFOX) can cause damage to the remaining liver. According to this study, regimens including OX are associated with an increased risk of vascular lesions (such as severe hepatic sinusoidal obstruction, also called ‘*blue liver*’), while regimens including IR have been associated with an increased risk of steatosis and steatohepatitis. Secondary analyses also show that the administration of six cycles of FOLFOX was associated with a moderate increase in the risk of reversible complications following surgery [12, 17].

Tol *et al* showed that the combination of capecitabin + IR (CAPIRI) was associated with a greater incidence of grade 3–4 toxicity, in comparison with the combination of 5-FU/LV + IR (FOLFIRI) [10]. In another study, pre-operative CT based on FOLFOX/FOLFIRI for LMCRC were not significantly associated with an increase in GS or PFD [18]. Nevertheless, the introduction of new therapeutic strategies made possible an increase in the expected GS for patients with non-resectable LMCRC to 22 months [19].

Although several mechanisms determine the action of conventional CT, they all seek to act on the cells, blocking the replication of their DNA. As they are not specific for the cancer cells, these mechanisms are often associated with toxicity for normal tissues [13, 20]. As a result, the specific biological molecular therapies for LMCRC have achieved great relevance in the last few years in the search for a more selective action [21]. The introduction of a new class or agents called monoclonal antibodies (MA) have provided additional benefits [10, 13, 20]. These agents bind to the binder (protein which binds to a receptor) or the extracellular domain of the receptor itself. That is this group of agents inhibits the means for the transduction of signals by means of the tyrosine kinase receptors which are necessary for the growth of the cancer cells (these have molecular targets present in their structure and microenvironment), thus blocking their development [10, 13, 20]. The identification of these agents is changing the treatment of LMCRC. Two types of MAs were approved by the US *Food and Drug Administration* (FDA) and by the *European Medicines Agency* (EMA) for clinical purposes in LMCRC: the endothelial vascular growth factor inhibitors (anti-VEGF), represented by bevacizumab; and the epidermal growth factor receptor inhibitors (anti-EGFR), represented by cetuximab and panitumumab [10, 14].

Cetuximab (Erbitux^®^) is a monoclonal chimeric G1 immunoglobulin (IgG1) which recognises and binds to the extracellular domain of the EGFR, blocking the activation of this receptor [13, 20, 22–24]. It was approved after an improvement in the survival and inverse chemoresistance was demonstrated when administered with IR in phase II trials [23], also presenting evidence of their efficacy and safety when added to FOLFIRI, as a first-line intervention for carriers of CCRm who have wild-type KRAS [24]. Approximately 20% of all colorectal cancers contain wild KRAS (the absence of a mutation on the KRAS oncogene) [25, 26, 27, 28] and only tumours which express the wild-type KRAS can be candidates for cetuximab, while the patients with mutant KRAS are considered resistant. It can be used as a monotherapy or in combination with primary, secondary, or tertiary configurations in patients with LMCRC [22].

The results of an analysis of the mutational state of KRAS done by the FIRE-3 study confirmed prior results showing that first-line CT (FOLFIRI) associated with cetuximab achieved a good response in terms of the global response rate and GS in the majority of CCRm carriers with wild KRAS (exon 2), extended to those with wild KRAS exons 3/4, and also wild NRAS oncogenes exons 2/3/4. Meanwhile, a subgroup of patients with mutated KRAS did not obtain a similar benefit, showing an improvement in PFD only following treatment with FOLFIRI associated with another MA. The results for PFD showed a similar tendency, patients with wild-type RAS and BRAF mutation obtained a discrete advantage with FOLFIRI associated with cetuximab, when compared with FOLFIRI alone, and when compared with FOLFIRI associated with another monoclonal antibody (FIRE III).

A recent meta-analysis which compared cetuximab + irinotecan versus chemotherapy alone, when used as a first-line treatment for LHCRC, demonstrated that the addition of cetuximab was statistically superior to isolated chemotherapy in terms of the general response to treatment, the GS time and the PFS [24]. Another study demonstrated that cetuximab can also preserve the quality of life (QOL) of patients with advanced CRC [29]. Among the most commonly observed adverse events in these studies were cutaneous toxicity (rash) [13, 24], reactions to the infusion, hypomagnesemia, fatigue, abdominal pain, nausea, and diarrhoea [13]. Among the inoperable LHCRC, the combinations of cetuximab + FOLFOX-6 (oxaliplatin/5-FU/LV) and cetuximab + FOLFIRI significantly increased the resectability of the metastases, including R0 resections [24].

Panitumumab (Vectibix^®^), a G2 kappa immunoglobulin and a human recombinant antibody, was established as an effective monotherapy in patients with LMCRC resistant to fluoropyrimidine, OX, and IR [14]. The results of two studies show that panitumumab improves the results when added to FOLFOX or FOLFIRI among patients with CCRm and wild-type KRAS [14]. Meanwhile, their application to mutated KRAS and NRAS genes (the latter a homologue of the viral RAS gene of the neuroblastoma) generates an inverse response, as these genes are capable of predicting the absence of the benefit from the addition of panitumumab to CT, (Douillard 2013). When administered as a first- or second-line treatment in combination with CT, panitumumab is shown to be significantly better than chemotherapy alone in terms of PFS in patients with metastatic colorectal cancer (MCRC) [30].

It has an acceptable tolerability profile when administered as a monotherapy or in combination with CT. Its toxicity is related to alterations in the skin and seems to present a low risk of immunogenicity [30]. Panitumumab received advance approval from the Food and Drug Administration (FDA) based on the improvement in PFS and an independent response rate of 8%, similar to that observed with other active agents in the advanced phase of the illness [31].

In a similar manner to panitumumab, clinical trials indicate that bevacizumab (Avastin^®^), a humanised recombinant antibody, in combination with 5-FU + folic acid and bevacizumab in combination with IR/5-FU/LV are clinically effective, when compared with standard first-line CT for the treatment of LMCRC [32]. These results seem to extend to second-line CT. A recent multicentric phase III study assessed the continued use of bevacizumab associated with second-line CT in patients with LMCRC who had progress after use of the drug with first-line CT [33]. The GS was statistically greater in the intervention group when compared with the group that received CT only [33]. Finally, Masi *et al* concluded that bevacizumab can be used safely with FOLFOXIRI (IR, OX, 5-FU, and folinate) after assessing the efficacy and safety of this plan in 57 patients with non-resectable LHCRC. The treatment achieved promising results in terms of PFS (equal to 74% at ten months (95% CI 62–85) [34].

Although there is evidence of the efficacy of this new class of drugs, the best pre-operative regimen is still not well established. Optimisation of results and reduction of toxicity are goals to be achieved [24]. The primary objective of this study is to assess the effectiveness and safety of MAs in the treatment of metastatic colorectal cancer, in addition to their effects.

## Methods

This study is a systematic review of the literature with meta-analysis. The following inclusion criteria were used: randomised clinical trials (RCTs) of adult participants (>18 years of age) of both sexes with a confirmed histological diagnosis of CRC and evidence of metastatic illness, assessing MAs associated with any CT (intervention group–at least one of the three drugs–cetuximab, bevacizumab, or panitumumab) versus CT without MAs (control group) or placebo, applied to metastatic illness.

We consider oral, systemic, or intravenous intervention associated with CT or anti-angiogenic agents (or even a combination of these treatment modalities) for the treatment of MCRC.

The criteria for exclusion were: other primary study types (quasi-randomised, cohort, and case-control clinical trials, among others); RCTs with participants <18 years of age; studies with volunteers; studies with a single branch of treatment; studies of non-pharmacological interventions (eg. surgery, radiation therapy).

The primary outcomes were GS and PFS, both measured in months. GS is understood as the time elapsed from the date of initial diagnosis of the metastasis and the date of death because of any cause [35]. PFS is the period of time from the randomisation to any progression of the illness or death, whichever happens first [15, 36]. We used the Response Evaluation Criteria in Solid Tumours (RECIST) version 1.1 [37] to define progression in this study, which was characterised as an increase of at least 20% in the major diameter of the target lesion, taking the lowest measurement of the major diameter, verified at the start of treatment or the appearance of one or more new lesions as a reference. The secondary objectives were the toxicity of the treatment, according to the Common Toxicity Criteria or the National Cancer Institute (NCI) of the United States, including the rate of deaths related to treatment. Other secondary objectives were: the time for progression, defined as the time from the randomisation and the objective tumour progression (not including deaths), which was also assessed according to the RECIST criteria; along with the rate of resectability, and the conversion rate.

The assessment of QOL represents an assessment of the impact of a health condition and its treatment on all of the pertinent and important aspects of the life of the patient, such as for example physical, social, psychological, symptoms, and perceptions.

### Search for identification of studies

**Figure 1.**
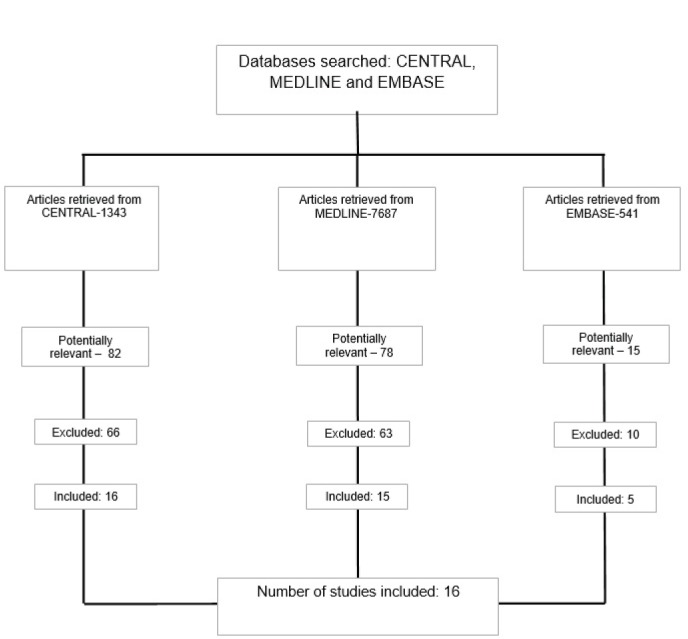
Study selection flowchart.

We identified all of the relevant studies (Figure 1), independently of year or language. RCTs were identified through a systematic search in the following electronic databases [Appendix 1]: *Cochrane Colorectal Cancer Group, The Cochrane Central Register of Controlled Trials* (CENTRAL); MEDLINE and EMBASE. The search for unpublished RCTs or those in progress was from their *metaRegister of Controlled Trials* [38] using the following search terms: monoclonal antibodies; mAb; anti-VEGF; anti-EGRF; bevacizumab OR, avastin OR. panitumumab OR. vectibix *cetuximab OR. erbitux OR. colorectal neoplasms OR. colorectal cancer OR. colorectal carcinomas*. The search was performed by two authors (Rosa BR, and Correia MM), independently and the concordance between them was assessed using the Kappa correlation coefficient. The last search was performed in March of 2014. The search strategy in PUBMED was the following: (‘Colorectal neoplasms/drug therapy’ [MESH] OR ‘Colorectal neoplasms/therapy [MESH]) AND (cetuximab [Title/Abstract] OR panitumumab [Title/Abstract]) OR, bevaczumab OR (CHEMOTHERAPY [Title/Abstract]).

### Analysis and synthesis

#### Assessment of the risk of bias in the studies included

**Figure 2.**
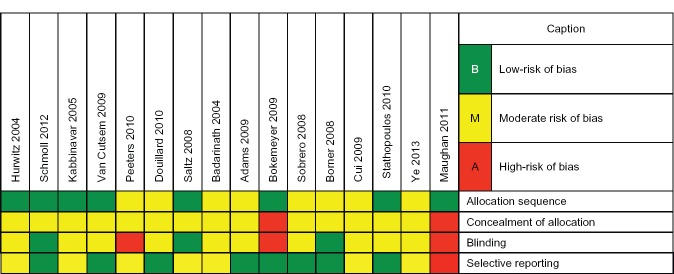
Summary of the risk of bias in the included studies.

The risk of bias in the studies included was assessed independently by means of a domain-based assessment, considering the following domains associated with the risk of bias: generation of the allocation sequence (method used to generate the allocation sequence); blinding of the allocation (method used for blinding the allocation sequence, in order to know if the allocations of interventions could be predicted in advance); blinding of participants, researchers, and assessors of results (means used to prevent the participants in the study from becoming aware of the intervention that they or any other participant may have received); and selective recording of results (a situation in which part of the study results may have been published). Each of these items was classified into categories: ‘Yes’, ‘No’, or ‘Uncertain’, indicating low, high, or unknown risk of bias, respectively. We contacted the authors of the studies when we found unclear or missing data. The results of these analyses are given in a chart of the risk of bias (Figure 2).

#### Measures of the effect or treatment, synthesis of data, and statistical heterogeneity among the studies

We compared the opposing data using the relative risk (RR) and the consistent data using the means difference. All of the estimates had a confidence interval of 95% (CI), where *p* < 0.05 was statistically significant. All of the analyses were done using the *Review Manager 5* (RevMan 5) software, from the *Cochrane* Team. Meta-analysis was done when at least one of the same outcomes was assessed in at least two RCTs. Analysis of the subgroup was done based on the KRAS mutation state (group 1: KRAS mutated; group 2: wild KRAS). Studies which included the two types of KRAS were not included in this type of analysis.

## Results

### Description of the studies and flow chart

The flow chart for the selection of the studies is shown in Figure 1. The reviewers finally agreed to include sixteen [16] published RCTs on the MAs in question, involving 12,015 participants and with a Kappa concordance coefficient of 0.5 (satisfactory) [39–54].

### Characteristics of the selected studies

These RCTs assessed cetxiumab (eight RCTs–5517 participants), bevacizumab (six RCTs–4129 participants) and panitumumab (two RCTs–2369 participants) associated with CT. Data from eight RCTs was included in the meta-analyses, with a total of 7128 participants. The median age of the participants was 61.2 years.

Cetuximab was assessed in eight studies (5517 participants) [44–49, 53, 54]. Of these, five added cetuximab to first-line CT. In two of these five, cetuximab was associated with OX + fluoropyrimidine versus the same strategy without MA, with a total of 2434 participants [44, 46]. While both had performed the studies with three branches of treatment, the analyses were considered in two groups. Another two studies (375 participants) assessed the role of cetuximab when associated with FOLFOX-4 (OX + leucovorin + 5-FU in a *bolus*) [47, 43]. The last added MA to FOLFIRI (irinotecan + leucovorin + 5-FU in an *infusion*), randomising the groups with or without cetuximab, with a total of 1198 participants [45]. Finally, one RCT assessed whether FOLFOX-6 associated with cetuximab would be more effective than FOLFIRI in 138 participants randomised into two groups [54]. In another study with 1298 participants for whom prior treatment with first-line CT had failed, (FOLFOX - OX + leucovorin + 5-FU (in an *infusion*)) or irinotecan (IR) was associated or not with cetuximab [48]. Another study used cetuximab + XELOX (OX + capecitabin) versus XELOX in 74 participants [49].

Bevacizumab was assessed in six RCTs (4129 participants) [39–41, 50–52]. In three of these (1097 participants), the combination of IR, 5-FU, and leucovorin (LV) were used with or without bevacizumab [39, 50, 51]. Two other studies used bevacizumab associated with different CT schemes [40, 41]. The first RCT (209 participants) compared bevacizumab to a placebo, both associated with the 5-FU + LV scheme [40]. The other study (1401 participants) [41] compared bevacizumab to a placebo, both associated with XELOX and FOLFOX-4 (bevacizumab + XELOX or FOLFOX-4 versus placebo + XELOX or FOLFOX-4). This study was initially performed with two treatment branches. Finally, the RCT which included 1422 participants compared bevacizumab to cediranib (an inhibitor of the tyrosine kinase of the vascular endothelial growth factor receptor), both combined with first-line CT [52]. Then panitumumab was assessed in two other studies totaling 2369 participants. In a study with 1186 participants this was associated or not with FOLFIRI [42].

### Risk of bias

Overall, the methodological quality of the studies was considered to be moderate. The allocation concealment method was not mentioned in any of 16 RCTs; it was the item with the highest risk of bias. Patients were not blinded in three of the eight RCTs that were included in the meta-analyses, totaling 3153 participants [42, 44, 47]. Randomisation was properly performed in eight RCTs; its status is unknown in the remaining eight studies. For this domain, the risk of bias was considered to be moderate. The practice of selective reporting was clearly identified in only one publication [44]. However, the CI of some means were not published in some RCTs, which prevented these studies from being included in the meta-analyses [39–42, 50, 52, 54].

Figure 3 presents the RCTs distribution in a funnel plot for OS (Figure 3A) and PFS (Figure 3B) outcomes. The funnel plot can indicate the presence of publication bias. A funnel plot is a scatter plot of the effect estimates of individual studies in some measure of size or precision of each study. In the absence of heterogeneity between studies, a possible dispersion will be because of sampling variation between clinical trials, and the graph may resemble a symmetrical inverted funnel. A triangle centered on the fixed effect estimation will extend for 1.96 standard error on each side and will include 95% of the studies if no bias is present under the assumption of fixed effect (which is the true treatment effect in each study). Otherwise, there may be asymmetry, which will indicate some sort of heterogeneity (statistical, methodological, or clinic) and/or publication bias.

### Excluded studies

After all the initial exclusions, 65 studies were classified as potentially relevant [55–119]. However, in a more rigorous evaluation, these studies did not match the inclusion criteria of the present review and were discarded. Nineteen were excluded because they had only one treatment group [55–73], 31 had at least one MA in more than one treatment group [74–104], three did not have CT associated with the intervention [105–107], three were retrospective studies [108–110], two were not RCTs [111, 112], three were secondary data analysis [113–115], two were locally severe disease [116, 117], one did not include metastatic disease [118], and the final study assessed the intervention after metastatic surgery [119].

### Effect of interventions

**Figure 4.**
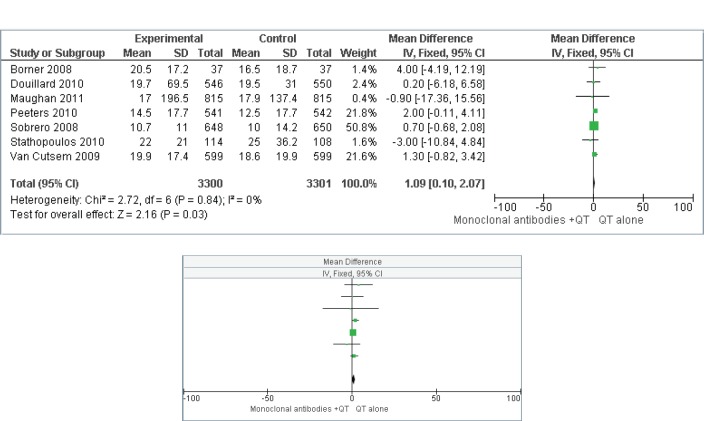
Overall survival (OS)–comparison: MAs + CT versus treatment without MAs.

**Figure 5.**
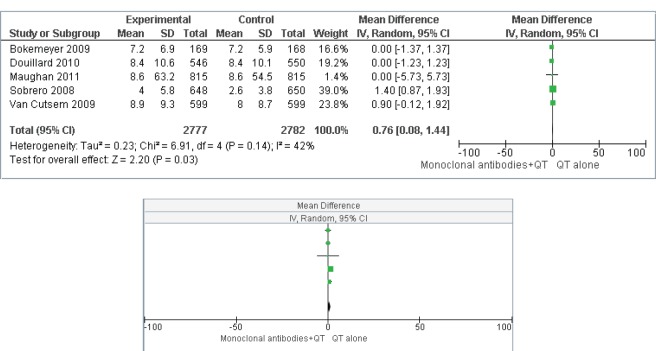
Progression-free survival(PFS)–comparison: MAs + CTversus treatment without MAs.

Figures 4 and 5 present the effect of interventions regarding OS (Figure 4) and PFS (Figure 5).

### Secondary outcomes

#### Toxicity and safety

Treatment toxicity (including the death rate), the proportion of patients eligible for resection, the time to progression, and the conversion rate were assessed in seven RCTs [39, 44, 46, 47, 52–54]. None of the above criteria could be assessed by meta-analysis, because the authors did not publish the CIs of the percentages and/or means for each of the groups. Treatment-related deaths were evaluated only in two RCTs (both with cetuximab), totaling 27 deaths among 2434 participants (1.1%), and 14 in control groups (CT only) versus 13 deaths in the intervention groups (CT + cetuximab) [44, 46]. Only one RCT reported no death by any cause [54].

### Resection rate

Metastatic resection only was evaluated in two studies [52, 54], both for liver metastases only. The first study reported a statistically significant difference among the 23 radical resections (R0–18 versus 5 - CT + cetuximab versus CT only, respectively–Odds ratio-4.37, P < 0.01) [54]. Conversely, the second study found no statistically significant difference on hepatic resection rate between treatment groups (4.4% in the control group (CT + cediranib) versus 5.2% in the CT + bevacizumab group—Odds ratio, 0,89; 95% CI- 0.54–1.46, P = 0.637) [52]. Because of the low percentage of resection cases, the logistic regression model was adjusted by treatment and by the criterion of single liver disease at baseline [52].

### Time to progression

Time to progression was assessed by a single study [49]. In that work, the time to disease progression was 7.2 months (95% CI: 6.0–8.4) for the intervention group (XELOX + cetuximab), versus 5.8 months (95% CI: 5.0–8.3) for the control group (XELOX only). In that case, although the author published the CI of the means, a meta-analysis was not performed, as it was the only study that assessed this outcome.

### Conversion rate and QOL

Conversion rate was not assessed by any of the RCTs. QOL was assessed in only two studies [39, 48].

### KRAS

#### Subgroup analysis of KRAS oncogene status

Five studies compared tumour response rate in subgroups stratified by KRAS status [42–45, 47]. However, one was excluded because although the author reported that PFS, response rate, and toxicity had been assessed in each group of KRAS status, the results of the mutated KRAS group were not published, which prevented any comparison and characterised selective reporting [44]. In the other studies, the groups with wild-type KRAS that received MA + CT had better results in every RCTs; in two of them, the difference was statistically significant [42, 45]. Conversely, for participants with mutated KRAS, the addition of MA to CT did not lead to better results when compared with CT alone (Table 1). Only one study (OPUS) conducted this type of evaluation regarding radical metastatic resection (R0) [47]. Similarly, patients with wild-type KRAS who received MA + CT had better results than those who have received CT only with twice the number of resections [47]. The author did not publish the statistical significance for this difference.

Again, the CI of the means were not published in those RCTs, which prevented the inclusion of these studies in the meta-analysis.

## Discussion

OS is a universally accepted hard outcome to assess cancer treatment results, and is the main outcome in the process of validation and approval of new drugs. PFS is not statistically validated as a surrogate outcome for survival in all scenarios; however, in the present study, there was no difference in response between the two groups when comparing the two types of outcome. For the OS outcome, the addition of MAs did not represent a protective factor, as the mean difference was 1.09 (CI 0.10–2.07). For the PFS outcome, the mean difference was 0.76 (CI 0.08–1.44), which indicates a health protection factor, although a statistically significant difference was not observed. An argument that could compromise these results would be the presence of heterogeneity between primary studies. However, this was considered to be at the best moderate. Although the funnel plots did not present dispersion, a range of 0–42% was observed when using the I2 statistic indicating respectively, low and moderate levels of inconsistency for OS and PFS.

When the analysis was focused on the type of KRAS, comparing the results of tumour response rate in participants who had the wild-type gene versus the mutated gene, the available results favoured wild-type KRAS (Table 1). KRAS is an oncogene, located on chromosome 6, which encodes a protein with an important physiological and pathogenic role in metastatic colorectal cancer (a key protein in EGFR signalling cascade). When certain mutations that may be present in tumours occur, especially in codons 12 and 13 of the KRAS gene, protein activation takes place regardless of the presence of EGFR, which is the target of some drugs for the treatment of this type of cancer, such as panitumumab and cetuximab. That is mutated KRAS has the potential to prevent the mechanism of action of the main drugs that treat metastatic colorectal cancer, which may explain the low results and the absence of a statistically significant difference observed in the mutated KRAS subgroup (Table 1).

Although this meta-analysis was based on high-quality methodologically correct RCTs, the present study had limitations. Its findings and interpretations were limited by the quality and quantity of information available. The non-inclusion of more recent and/or unpublished studies is a potential selection bias. Publication bias was the main problem observed, especially for conducting the analyses. The allocation concealment method was not determined in any of 16 RCTs included. Some authors did not publish the CI of the means of the outcomes of OS, PFS, and time to progression, which made the estimation of standard deviations (SD) and the inclusion of the RCTs in the metaanalysis impossible [39–42, 50, 52]. As they were drug studies, the nature of the evaluated interventions allows for blinding which would reduce the likelihood of performance bias.

Trials with various experimental groups can pose a potential problem. Many studies are designed to compare more than two treatments. In these cases, there may be two or more experimental interventions, for instance, a drug in different doses, or variations in the therapeutic schemes associated with that intervention. It may be inappropriate to combine heterogeneous intervention groups and compare them with the control group, since it will impair the confidence regarding that result. When there is only one control group, it could be divided; however, to combine these results in the same meta-analysis is inappropriate, because they will be counted twice.

According to *The Grades of Recommendation, Assessment, Development and Evaluation Working Group* (GRADE) [120] an evidence quality rating system, the estimate of the effect decreases if the studies present severe limitations in their design and implementation that can result in a partial assessment of the effects of the intervention. For RCTs, these methodological limitations include important issues such as lack of confidentiality in the allocation, lack of blinding (especially in studies with subjective outcomes that are highly susceptible to biased evaluation), large losses to follow-up, early study termination, or even selective reporting of results among others. Grade is an important tool that defines the quality of a body of evidence as a measure that ensures that an effects or association estimation is close to the specific quantity of interest.

In the early 1980s, the FDA determined that drugs approved for the treatment of cancer should be based on more direct evidence of clinical benefit, such as improvement in survival, in the QOL, or in the symptoms related to the tumour. This study aimed to verify such evidence for MAs in the treatment of metastatic colorectal cancer. Although this study included only patients with metastatic disease, Maughan’s RCT (2011) [44] was included in the analysis, as only 2% of that sample (13 patients) did not have metastasis at any site. The selected studies did not allow for conversion analysis, in total nor in the KRAS status subgroup.

## Conclusion

In conclusion, this meta-analysis demonstrated that the addition of MAs (bevacizumab, panitumumabe, or cetuximab) to OX or irinotecanbased chemotherapy schemes in first-line treatment in patients with resectable liver metastasis did not increase OS or PFS. Because of the heterogeneity of the studies, particularly regarding KRAS status, prospective controlled clinical trials to study the combination of drugs are needed.

## Conflicts of interest

The authors declare no conflicts of interest.

## Figures and Tables

**Figure 3A.**
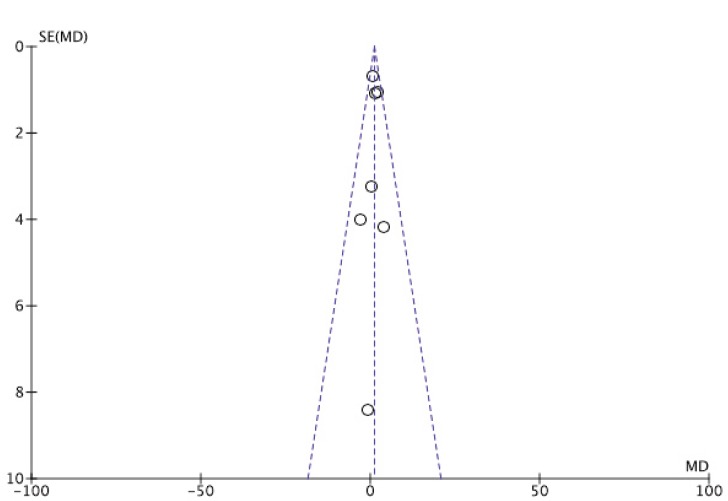
Funnel plot of overall survival (OS).

**Figure 3B.**
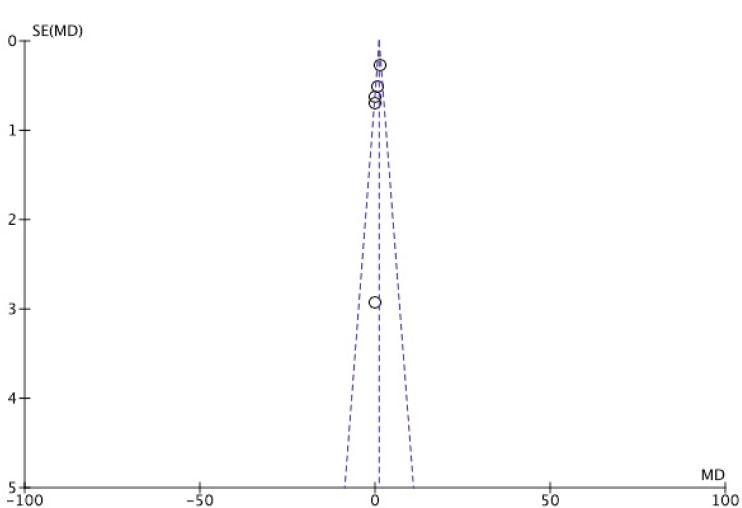
Funnel plot of progression-free survival (PFS).

**Table 1.** Subgroup analysis by KRAS status regarding tumour response rate.

Study	Response rate (%)							
	Wild-type KRAS		Odds ratio	P	Mutated KRAS		Odds ratio	P
	MA + CT	CT			MA + CT	CT		
Peeters^38^	35	10	–	**0.001**	13	14	–	–
Douillard^39^	55	48	1.35	0.068	40	40	–	–
Van Cutsem^41^	59.3	43.2	1.91	**0.03**	36.2	40.2	0.80	–
Bokemeyer^43^	3	1	2.54	0.11	0	2	0.50	0.106
